# The effects of oral antioxidants on outcomes of assisted reproductive technology: a systematic review and meta-analysis of randomized controlled trials

**DOI:** 10.3389/fendo.2026.1891276

**Published:** 2026-07-22

**Authors:** Ruonan Qiang, Zheyu Xu, Yingqiao Wang, Yuanye Gu, Yanfeng Liu, Qifan He, Yinan Zhu, Xue Pan

**Affiliations:** 1 Dongzhimen Hospital, Beijing University of Chinese Medicine, Beijing, China; 2 Longhua Hospital Shanghai University of Traditional Chinese Medicine, Shanghai, China; 3 Third Affiliated Hospital, Beijing University of Chinese Medicine, Beijing, China

**Keywords:** antioxidants, assisted reproductive technology, IVF/ICSI-ET, meta-analysis, oxidative stress, randomized controlled trial

## Abstract

**Background:**

The clinical efficacy of antioxidants in assisted reproductive technology (ART) remains inconsistent. This systematic review and meta-analysis aimed to evaluate the impact of oral antioxidant supplementation on reproductive outcomes in patients undergoing *in vitro* fertilization/intracytoplasmic sperm injection-embryo transfer (IVF/ICSI-ET).

**Methods:**

We systematically searched PubMed, Scopus, Embase, and Web of Science databases from inception to February 2026 for randomized controlled trials (RCTs) investigating oral antioxidants in patients undergoing ART. We performed meta-analyses by pooling effect sizes, conducting subgroup analyses, and exploring heterogeneity. Methodological quality was assessed using the Risk of Bias (RoB) tool, and the certainty of evidence was evaluated using the Grading of Recommendations Assessment, Development, and Evaluation (GRADE) framework. Sensitivity analyses and publication bias assessments were also conducted.

**Results:**

This meta-analysis suggests that oral antioxidant supplementation may provide potential clinical benefits for women undergoing IVF/ICSI-ET. The pooled estimates indicated a significant improvement in the clinical pregnancy rate (RR = 1.29, 95% CI: 1.070–1.554), the number of morphologically mature oocytes (MII oocytes) (MD = 2.99, 95% CI: 1.82–4.16), the number of high-quality embryos (MD = 1.08, 95%CI: 0.69–1.47), and the live birth rate (RR = 1.407, 95% CI: 1.052–1.881). Due to the limited number of included studies, further large-scale RCTs are required to confirm these findings regarding high-quality embryos, live birth rates, and miscarriage rates, thereby strengthening the overall level of evidence.

**Conclusion:**

Oral antioxidant supplementation as an adjuvant therapy shows potential clinical benefits in improving clinical pregnancy rates, MII oocyte yields, the number of high-quality embryos, and live birth rates in women undergoing IVF/ICSI-ET. Notably, antioxidants appear to offer broader clinical utility in patients undergoing the antagonist protocol. Given the current limitations in study volume and methodological heterogeneity, future well-designed RCTs—stratified by patient characteristics and employing standardized antioxidant regimens—are warranted. In particular, studies prioritizing live birth as the primary endpoint are essential to establish definitive clinical recommendations.

**Systematic review registration:**

identifier CRD420251238213.

## 1 Introduction

The World Health Organization (WHO) reports that infertility is a major global health challenge, with a prevalence of approximately 17.8% in high-income countries and 16.5% in low-income countries, meaning that roughly one in six people worldwide is affected by infertility (1). Assisted reproductive technology (ART), as one of the most significant paradigm shifts in the reproductive field, has provided immense support in responding to demographic changes, treating infertility, enhancing fecundity, and improving pregnancy outcomes (2). ART are defined as all interventions that include the collection of gametes (e.g., through ovarian stimulation and/or the surgical removal of oocytes), *in vitro* manipulation, and reproduction techniques such as *in vitro* fertilization-embryo transfer (IVF-ET), intracytoplasmic sperm injection-embryo transfer (ICSI-ET), trophectoderm biopsy, and preimplantation genetic testing (PGT), as well as embryo cryopreservation (2). The cornerstone of ART success lies in the acquisition of high-quality oocytes and embryos, along with favorable endometrial receptivity. Despite the rapid advancement of ART, its success rate remains relatively limited. In addition, with the increasing prevalence of infertility and the rising demand for pregnancy among women of advanced reproductive age, ensuring the safety, efficacy, and accessibility of ART treatment has consistently been a central focus in the field of reproductive medicine (3).

Oxidative stress (OS) is a condition characterized by the disruption of the balance between the production of reactive oxygen species (ROS) and the antioxidant defense mechanisms that maintain cellular redox homeostasis. It profoundly affects oocyte development, cryopreservation success rates, embryo quality, and implantation, and is a major contributor to diminished oocyte quality, reduced endometrial receptivity, and recurrent embryo transfer failure (4–6). Moreover, advancing age is associated with elevated OS levels, which has also been demonstrated to be a key factor in reproductive aging, declining fertility, and ART failure (6). Among these, the impact of OS on oocytes is particularly profound and exerts a dual role: tightly regulated redox signaling is essential for folliculogenesis and ovulation, whereas excessive ROS can affect the oocyte in terms of meiotic progression, spindle integrity, chromosomal aneuploidy, DNA damage, apoptosis, and cytoskeleton structure (7). Antioxidants such as coenzyme Q10, melatonin, and nicotinamide are increasingly being demonstrated to not only scavenge ROS but also modulate mitochondrial function, inflammatory responses, autophagy, and apoptosis, which are critical for ameliorating OS-related reproductive health issues (8).

In recent years, although multiple clinical studies have confirmed the considerable potential of antioxidants in improving female reproductive function and enhancing ART success rates, the evidence regarding the effect of antioxidants on ART reproductive outcomes remains insufficient due to variations in administration routes, study designs, and outcome measures (4). Most existing systematic reviews focus on a specific antioxidant, yield inconsistent conclusions, or include observational studies which may have high risk of bias and fail to adequately control for confounding factors, making it difficult to draw definitive conclusions. Currently, a systematic review of randomized controlled trials (RCTs) evaluating the effect of antioxidants on ART reproductive outcomes is still lacking, and several recent RCTs have provided relevant reports. Therefore, this systematic review and meta-analysis aims to evaluate the effect of antioxidant supplementation on IVF/ICSI-ET-assisted reproductive outcomes, thereby potentially providing a reference for the clinical application and basic research of antioxidants in the field of assisted reproduction.

## 2 Methods

### 2.1 Protocol and registration

This systematic review and meta-analysis was registered on the PROSPERO website (CRD420251238213). It was designed and conducted in accordance with the Preferred Reporting Items for Systematic Reviews and Meta-Analyses (PRISMA) 2020 recommendations for systematic reviews and meta-analyses (9) and adhered to the PRISMA guidelines presented in **Supplementary Table 1**. The review focuses on the effect of oral antioxidant supplementation, compared with placebo or standard care, on assisted reproductive technology outcomes.

### 2.2 Literature search

A systematic search was conducted in four major electronic databases: PubMed, Scopus, Embase, and Web of Science. The search was initially performed on February 10, 2026, with a confirmatory update on March 26, 2026, and was restricted to articles published in English. The search strategies employed keyword combinations tailored to each database to ensure a comprehensive retrieval of relevant literature. The detailed search terms are provided in **Supplementary Tables 2**–**S4**.

### 2.3 Study selection

The study selection process was conducted independently by two reviewers—R.Q. and Z.X.—based on predetermined inclusion and exclusion criteria to ensure the relevance and quality of the included studies. First, R.Q. and Z.X. independently screened the titles and abstracts of all retrieved articles. Any discrepancies were resolved through confirmation and discussion with a third reviewer (Y.L.), completing the initial screening phase. Subsequently, Y.L. conducted a comprehensive assessment of all potentially eligible studies identified in the initial screening to ensure the comprehensiveness and precision of the final included articles. The entire process was managed using EndNote 2025 software.

Studies meeting the following criteria were included (1): RCTs evaluating the effect of antioxidants on assisted reproductive technology outcome measures; (2) antioxidant supplementation administered orally to women; (3) ART cycles restricted to IVF or ICSI interventions; (4) studies reporting at least one of the following: clinical pregnancy rates; the number of morphologically mature oocytes (MII oocytes); the number of high-quality embryos; live birth rate; miscarriage rate, or other indicators reflecting assisted reproductive technology outcomes; (5) control group receiving placebo or non-antioxidant standard care; (6) provision of sufficient quantitative data for meta-analysis; (7) full-text articles published in English.

Studies were excluded if they met any of the following criteria: (1) non-randomized, observational studies, case reports, animal studies, or studies solely based on *in vitro* experiments; (2) lack of a placebo/non-antioxidant standard care control group, or control group containing antioxidants, or evaluation of antioxidants used in combination with other drugs where the effect of antioxidants could not be isolated, or antioxidant complex preparations containing non-antioxidant ingredients, or absence of a suitable control group to isolate the antioxidant effect; (3) lack of assisted reproduction-related outcome measures or insufficient quantifiable data; (4) non-English publications or conference abstracts, narrative reviews, editorials, retracted studies, and other studies without accessible full-text. **Table 1** presents the PICOS (Population, Intervention, Comparison, Outcomes, and Study) framework and research question for this review.

**Table 1:** PICOS framework and research question for this review.

PICO element	Description
Population (P)	Infertile women undergoing IVF/ICSI-ET.
Intervention (I)	Oral antioxidant supplementation.
Comparison (C)	Placebo or standard treatment without antioxidant supplementation.
Outcomes (O)	Clinical pregnancy rates; the number of MII oocytes; the number of high-quality embryos; live birth rate; miscarriage rate.
Study design (S)	Randomized controlled trials.

### 2.4 Data extraction

Data were extracted independently by two reviewers using a pre-specified standardized form. Extracted information included study characteristics (first author, publication year, country, study design), participant characteristics (sample size, mean age, patient population, BMI, duration of infertility), intervention details (antioxidant, dosage, ovarian stimulation protocol), control group specifications (type of control, placebo dosage if applicable), and outcome data (clinical pregnancy rates; the number of MII oocytes; the number of high-quality embryos; live birth rate; miscarriage rate). Discrepancies were resolved through discussion or consultation with a third reviewer.

### 2.5 Quality assessment and GRADE assessment

Two reviewers (R.Q. and Z.X.) independently assessed the methodological quality of the included studies using the Cochrane risk-of-bias tool for randomized trials (RoB 2), evaluating seven domains: random sequence generation, allocation concealment, blinding of participants and personnel, blinding of outcome assessment, incomplete outcome data, selective outcome reporting, and other sources of bias. Each domain was rated as low, unclear, or high risk of bias. Studies were classified as high quality if most domains were at low risk of bias, moderate quality if one or two domains were at high risk, and low quality if multiple domains were at high risk. The certainty of evidence was evaluated using the Grading of Recommendations, Assessment, Development, and Evaluation (GRADE) approach, facilitated by the Cochrane GRADEpro GDT online software platform (www.gradepro.org). Discrepancies in assessment were resolved through consensus or discussion with a third reviewer (Y.L.) to ensure the accuracy and consistency of the study assessments.

### 2.6 Data synthesis and statistical analysis

OS exerts detrimental effects on female reproductive function and is recognized as a key mechanism underlying decreased oocyte quality, reduced yield of high-quality embryos, and suboptimal ART success rates. This systematic review and meta-analysis aimed to evaluate the impact of antioxidant supplementation as an adjuvant strategy on reproductive outcomes in women undergoing IVF/ICSI-ET. Although the included studies utilized diverse antioxidants—varying in dosage, duration, and timing—and covered a spectrum of patient characteristics and ovarian stimulation protocols, all interventions shared a unifying biological rationale: the attenuation of OS to improve reproductive function (specifically oocyte and embryo quality). To address the potential impact of clinical heterogeneity on the robustness of our pooled effect estimates, we conducted heterogeneity testing for each outcome, employed appropriate effect models for data synthesis, and performed sensitivity analyses. For outcomes exhibiting high heterogeneity, we further investigated potential sources using subgroup analyses and Baujat plots, interpreted alongside our sensitivity analysis results. While our findings may not reflect the efficacy of any single compound or specific regimen, they provide an assessment of the overall therapeutic trend, offering valuable insights to guide future research on antioxidant applications in ART.

Statistical analysis for this systematic review was performed mainly using the “meta”, “metafor”, and “dplyr” packages within the R software environment. Given that all included studies were RCTs, dichotomous outcomes were expressed as risk ratio (RR) with its 95% confidence interval (CI), whereas continuous outcomes were evaluated using mean difference (MD) and its corresponding 95% CI as the effect measures. To prevent the artificial inflation of weight and inaccurate pooled estimates stemming from the double-counting of a shared placebo arm in multi-arm trials investigating multiple antioxidant dosages, we adopted a consolidated group strategy (10). For continuous endpoints (such as baseline maternal age, the number of MII oocytes, and the number of high-quality embryos), the pooled mean and standard deviation (SD) were synthesized using standard formulae based on sample sizes, means, and intra-group variances of the respective arms. For dichotomous outcomes and baseline variables (clinical pregnancy rates, live birth rate, and miscarriage rate), the number of events and the total sample size across all multi-dose melatonin groups were directly summed. The pooled antioxidant group was subsequently compared against the control group in the primary analysis. The specific computational formulae were as follows:

Combined sample size: $N_{combined}=\sum_{i=1}^{k}N_{i}$

Combined mean: ${\bar{X}}_{combined}=\frac{\sum_{i=1}^{k}(N_{i}\times {\bar{X}}_{i})}{N_{combined}}$

Combined standard deviation: $SD_{combined}=\sqrt{\frac{\sum_{i=1}^{k}(N_{i}-1)SD_{i}^{2}+\sum_{i=1}^{k}N_{i}{\left({\bar{X}}_{I}-{\bar{X}}_{combined}\right)}^{2}}{N_{combined}-1}}$

A random-effects model was applied when heterogeneity was detected. Otherwise, a fixed-effect model was implemented for the analysis. Heterogeneity was assessed using the I² statistic and the Cochran Q test (*P* < 0.10 indicating significant heterogeneity). Sources of heterogeneity were explored through sensitivity analyses, and subgroup analyses (stratified by participant characteristics such as the type of antioxidants, disease, and stimulation protocol) to identify potential effect modifiers. For outcomes exhibiting substantial heterogeneity, a Baujat plot was generated to further investigate the potential sources of variation. For outcomes incorporating a sufficient number of eligible studies, a leave-one-out sensitivity analysis was executed to evaluate the robustness of the meta-analytic findings. Publication bias was evaluated using funnel plots and Egger’s test (*P* < 0.05 for significance). All synthesized outcomes were visualized using forest plots, where the horizontal span of each data point represents the 95% CI of the pooled effect size after omitting that specific study. A significance level of *p* < 0.05 was applied to all tests except the Q test.

## 3 Results

### 3.1 Search outcomes

**Figure 1:**
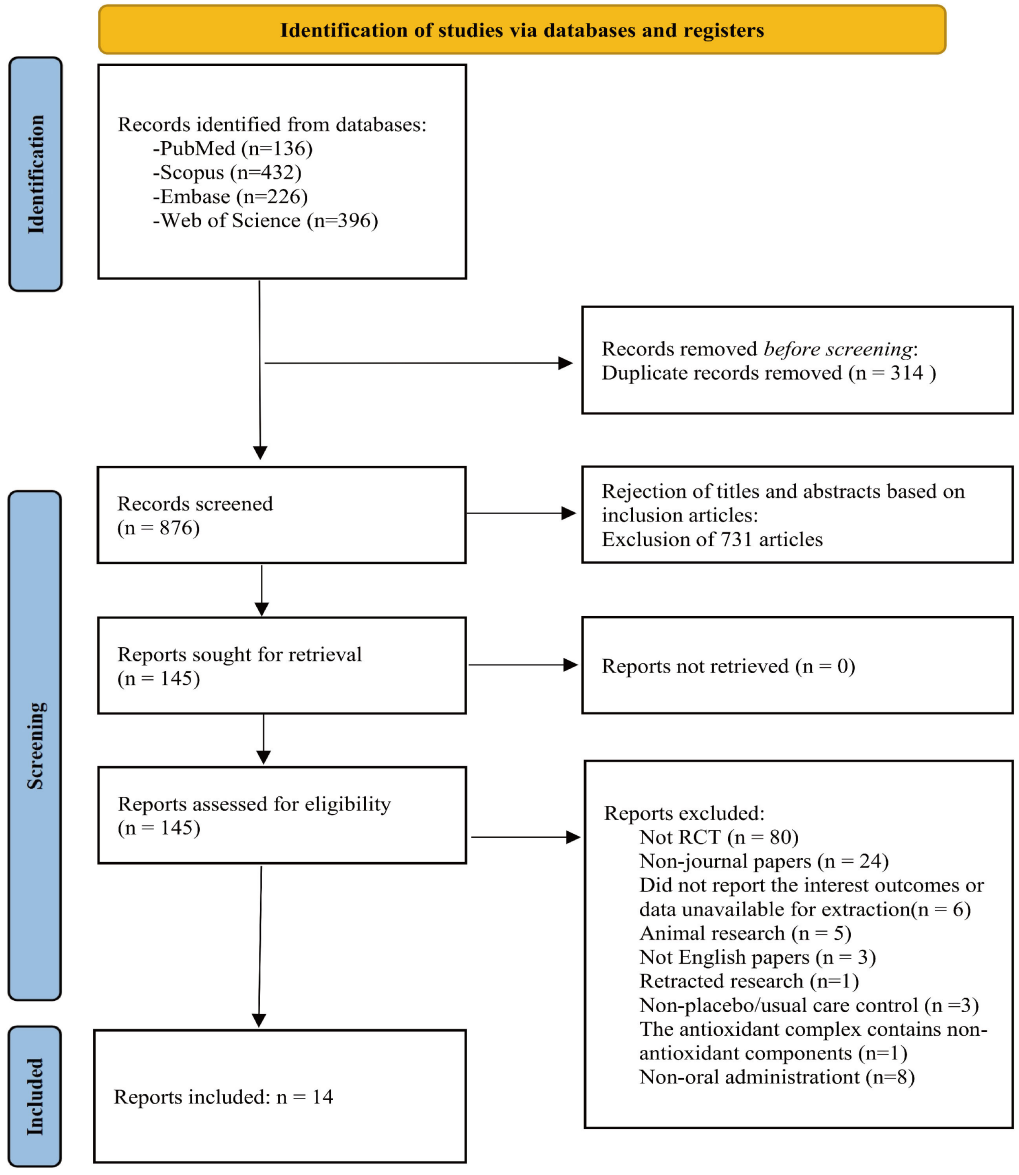
Flow diagram for study selection process.

**Figure 1** summarizes the study selection process. Initially, 1190 records were identified from databases and registers, including PubMed (n = 136), Scopus (n = 432), Embase (n = 226), and Web of Science (n = 396). After removing duplicates identified using EndNote software (n = 314), 876 unique records were screened based on titles and abstracts. Of these, 731 records were excluded because they did not meet the eligibility criteria, primarily because the study topic or outcomes were not relevant to this review. The remaining 145 studies were subjected to full-text assessment. Among these, 80 were excluded due to non-randomized design, 24 were non-journal articles, 6 did not report relevant outcomes or data unavailable for extraction, 5 were animal studies, 3 were non-English papers, 1 was a retracted study, 3 lacked a placebo/standard care control, 1 was an antioxidant complex containing non-antioxidant components, and 8 were non-oral administration. Ultimately, 14 RCTs of oral antioxidant supplementation met all eligibility criteria and were included in this systematic review and meta-analysis.

### 3.2 Baseline characteristics of included studies

A total of 14 RCTs comprising 1190 participants (intervention group: n = 637; control group: n = 553) were included in this meta-analysis. The mean maternal age ranged from 29.38 ± 5.52 to 38.42 ± 1.79 years in the intervention arms, and from 27.9 ± 2.8 to 38.32 ± 1.67 years in the control arms. The mean BMI spanned from 21.85 ± 2.51 to 28.91 ± 4.52 kg/m² for the intervention groups, and from 22.24 ± 3.07 to 27.72 ± 5.47 kg/m² for the control groups. Investigated antioxidant variants included melatonin, N-Acetylcysteine, coenzyme Q10, astaxanthin, resveratrol, nanomicelle curcumin. The targeted patient populations encompassed polycystic ovary syndrome (PCOS), poor ovarian response (POR), unexplained infertility, diminished ovarian reserve (DOR), not reported, women with advanced age, endometriosis, and sleep disturbances. Covered ART cycles involved IVF-ET, ICSI-ET, or a combination of both. The implemented controlled ovarian stimulation (COS) protocols consisted of the long protocol (GnRH agonist) and the antagonist protocol. All 14 trials adopted an RCT design, with 9 being placebo-controlled. Regarding outcomes, 12 studies reported clinical pregnancy rates, 9 recorded the number of MII oocytes, 4 quantified the number of high-quality embryos (defined as grade); 3 provided live birth rates (with one reporting ongoing pregnancy rate), and 2 provided data on miscarriage rates. Detailed study characteristics are presented in **Table 2**.

**Table 2:** Characteristics of included studies.

Author(s)	Study design	Country	ART	Disease	Randomized samplesize(EG)	Randomized samplesize(CG)	Analyzed samplesize(EG)	Analyzed samplesize(CG)	Age EG	Age CG	BMI EG	BMI CG	Infertility duration (years) EG	Infertility duration (years) CG	Stimulation protocol	Antioxidants	Intervention of EG	Intervention of CG	Duration	Outcomes
Batıoğlu AS, 2012 (11)	RCT	Turkey	IVF-ET	Not reported	40	45	40	45	30.4 ± 5.6	29.7 ± 4.9	Not reported	Not reported	Not reported	Not reported	Long protocol (GnRH agonist)	Melatonin	3 mg melatonin daily	Without melatonin treatment	Not reported	①②
Cheraghi E, 2016 (12)	RCT	Iran	ICSI-ET	PCOS	20	20	15	15	29.67 ± 3.35	27.9 ± 2.8	27.7 ± 4.5	26.9 ± 2.3	Not reported	Not reported	Long protocol (GnRH agonist)	N-acetylcysteine (NAC)	NAC 600 mg three times daily	Placebo-treate (oral rehydration solution)	6 weeks	①②③
Xu Y, 2018 (13)	RCT	China	ICSI/IVF-ET	Poor ovarian response	93	93	76	93	32.50 ± 3.30	31.92 ± 3.68	21.85 ± 2.51	22.24 ± 3.07	3 (2, 4)	3 (2, 3)	Antagonist protocol	Coenzyme Q10	Oral administration of CoQ10 200 mg three times a day	Without any additional treatment	60 days	①④⑤
Espino J, 2019 (14)	RCT	Spain	ICSI-ET	Unexplained infertility	20=10(3mg melatonin group)+10(6mg melatonin group)	10	20=10(3mg melatonin group)+10(6mg melatonin group)	10	35.48 ± 2.90	35.93 ± 3.20	23.54 ± 3.07	23.83 ± 3.53	Not reported	Not reported	Antagonist protocol	Melatonin	3 mg or 6 mg melatonin daily	Without melatonin treatment	40 days	①
Gharaei R, 2022 (15)	RCT	Iran	ICSI-ET	PCOS	21	21	20	20	30.60 ± 4.98	30.45 ± 3.98	28.91 ± 4.52	27.72 ± 5.47	Not reported	Not reported	Antagonist protocol	Astaxanthin (AST)	AST 8 mg (2 × 4 mg capsules) daily	Oral placebo capsules daily	40 days	①
Li X, 2022 (16)	RCT	China	ICSI/IVF-ET	Women with advanced age	100	100	100	100	37.83 ± 2.09	37.81 ± 1.79	24.03 ± 2.84	24.08 ± 2.96	4.04 ± 3.11	4.03 ± 2.74	Antagonist protocol	N-acetylcysteine (NAC)	NAC 0.6 g three times daily	Without NAC treatment	Nearly 45 days	①④⑤
Rostami S, 2023 (17)	RCT	Iran	ICSI-ET	Endometriosis	28	29	25	25	33.33±4.97	32.08 ± 5.09	23.17 ± 1.82	24.31 ± 2.60	3.56 ± 3.19	2.96 ± 1.54	Antagonist protocol	Astaxanthin (AST)	6 mg oral AST daily	6 mg oral placebo capsules daily	12 weeks	①②③
Ardehjani NA, 2024 (18)	RCT	Iran	ICSI-ET	PCOS	28	28	24	24	29.38 ± 5.52	30.75 ± 5.17	26.38 ± 3.04	26.48 ± 4.01	5.04 ± 2.35	5.29 ± 2.77	Antagonist protocol	Resveratrol	800 mg (2 × 400 mg capsules) daily	Placebo capsules (maltodextrin and lactose) daily	60 days	①②
Shafie A, 2024 (19)	RCT	Iran	ICSI-ET	Poor ovarian response	30	30	26	25	38.42 ± 1.79	38.32 ± 1.67	22.59 ± 1.25	22.46 ± 1.53	3.31 ± 1.25	2.88 ± 1.42	Antagonist protocol	Astaxanthin (AST)	12 mg of oral AST capsules per day (3 × 4 mg capsules)	Three placebos capsules (containing edible paraffin)	8 weeks	①②③
Jannatifar R, 2025 (20)	RCT	Iran	ICSI-ET	Endometriosis	25	25	25	25	32.6 ± 3.8	31.1 ± 3.3	23.6 ± 3.2	24.18 ± 2.13	5.46 ± 1.19	6.13 ± 1.44	Antagonist protocol	Nanomicelle curcumin	120 mg of oral nanomicelle curcumin daily	Placebo capsules daily	10 weeks	②
Sadeghpour S, 2025 (21)	RCT	Iran	IVF-ET	Diminished ovarian reserve	42	42	35	33	34.56 ± 4.69	34.73 ± 5.04	Not reported	Not reported	Not reported	Not reported	Long protocol (GnRH agonist)	Melatonin	3 mg melatonin daily	Placebos daily	Starting on the 3-5th day of the previous menstrual cycle until the oocyte retrieval.	②
Eryilmaz OG, 2011 (22)	RCT	Turkey	IVF-ET	Sleep disturbances	30	30	30	30	30.1 ± 4.0	29.9 ± 3.6	25.7 ± 4.5	26.1 ± 3.5	7.5 ± 3.9	6.4 ± 3.7	Long protocol (GnRH agonist)	Melatonin	3 mg melatonin daily	Without melatonin treatment daily	From the 3rd to the 5th day of the menstrual cycle until the human chorionic gonadotropin (hCG) injection day of the controlled ovarian hyperstimulation.	①②
Fernando S, 2018 (23)	RCT	Australia	ICSI/IVF-ET	Not reported	120=41(2mg melatonin group)+39(4mg melatonin group)+40(8mg melatonin group)	40	120=41(2mg melatonin group)+39(4mg melatonin group)+40(8mg melatonin group)	40	35.46 ± 4.22	35.2 ± 4.2	24.6 ± 4.1	24.5 ± 4.8	Not reported	Not reported	Antagonist protocol	Melatonin	2mg/4mg/8mg melatonin capsule twice per day	Placebo capsules twice per day	From day 2 of their cycle until the night before oocyte retrieval	①④
Jahromi BN, 2017 (24)	RCT	Iran	IVF-ET	Diminished ovarian reserve	40	40	32	34	35 ± 5.1	35.1 ± 5.1	Not reported	Not reported	6 ± 4.6	7.05 ± 5.7	Long protocol (GnRH agonist)	Melatonin	3 mg melatonin capsule daily	Placebo capsules daily	Approximately 1.5–2 menstrual cycles (from day 5 of the preceding cycle until oocyte retrieval).	①②③

①clinical pregnancy rates; ②the number of morphologically mature oocytes (MII oocytes); ③the number of high-quality embryos; ④live birth rate; ⑤ miscarriage rate. Study [(Li X, 2022) (16)] reported only OPR without live birth data were included in the live birth meta-analysis. ④Ongoing pregnancy rate (OPR) (ultrasound-confirmed viable intrauterine pregnancy ≥12 weeks) served as a valid surrogate for live birth, with high positive predictive value. IVF-ET, in vitro fertilization-embryo transfer; ICSI-ET, intracytoplasmic sperm injection-embryo transfer; PCOS, polycystic ovary syndrome.

### 3.3 Risk of bias assessment

**Figure 2:**
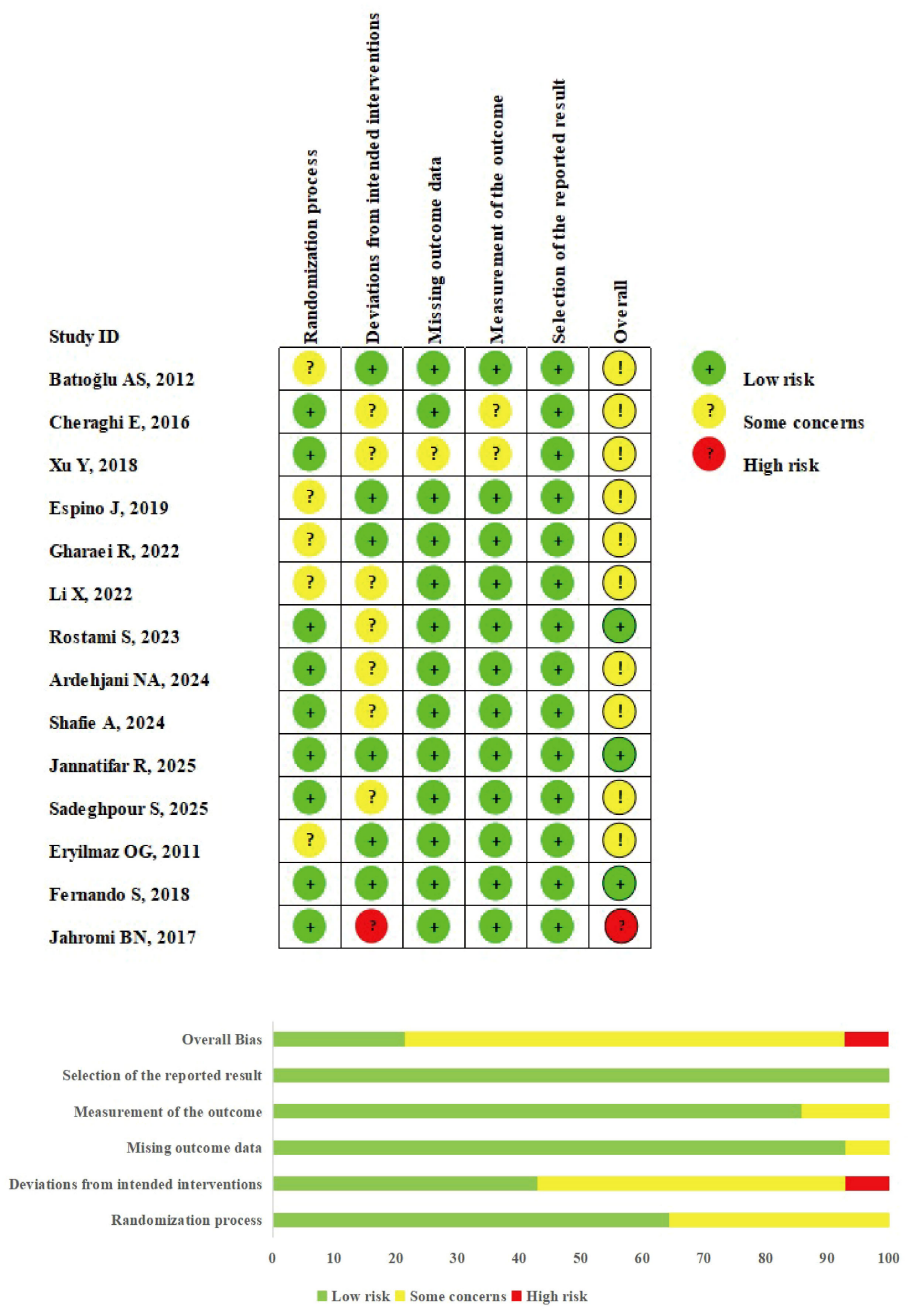
Risk of bias summary: review author’s judgements about each risk of bias item for included studies.

The methodological quality assessment indicated that most of the included literature strictly adhered to the design principles of randomized controlled trials. All 14 studies described their methods for random sequence generation (11–24), and the intervention and control groups demonstrated excellent baseline comparability. However, 5 trials were graded as having “some concerns” in the “randomization process” domain owing to a lack of explicit information regarding allocation concealment (11, 14–16, 22). One study was judged to be at high risk due to the absence of intention-to-treat (ITT) or modified intention-to-treat (mITT) analyses, which was likely to have substantially influenced the reported outcomes (24). Collectively, the quality of the included literature met the scientific thresholds, and the overall risk of bias was deemed acceptable. Detailed assessment results are shown in **Figure 2**.

### 3.4 Primary outcomes

#### 3.4.1 Clinical pregnancy

**Figure 3:**
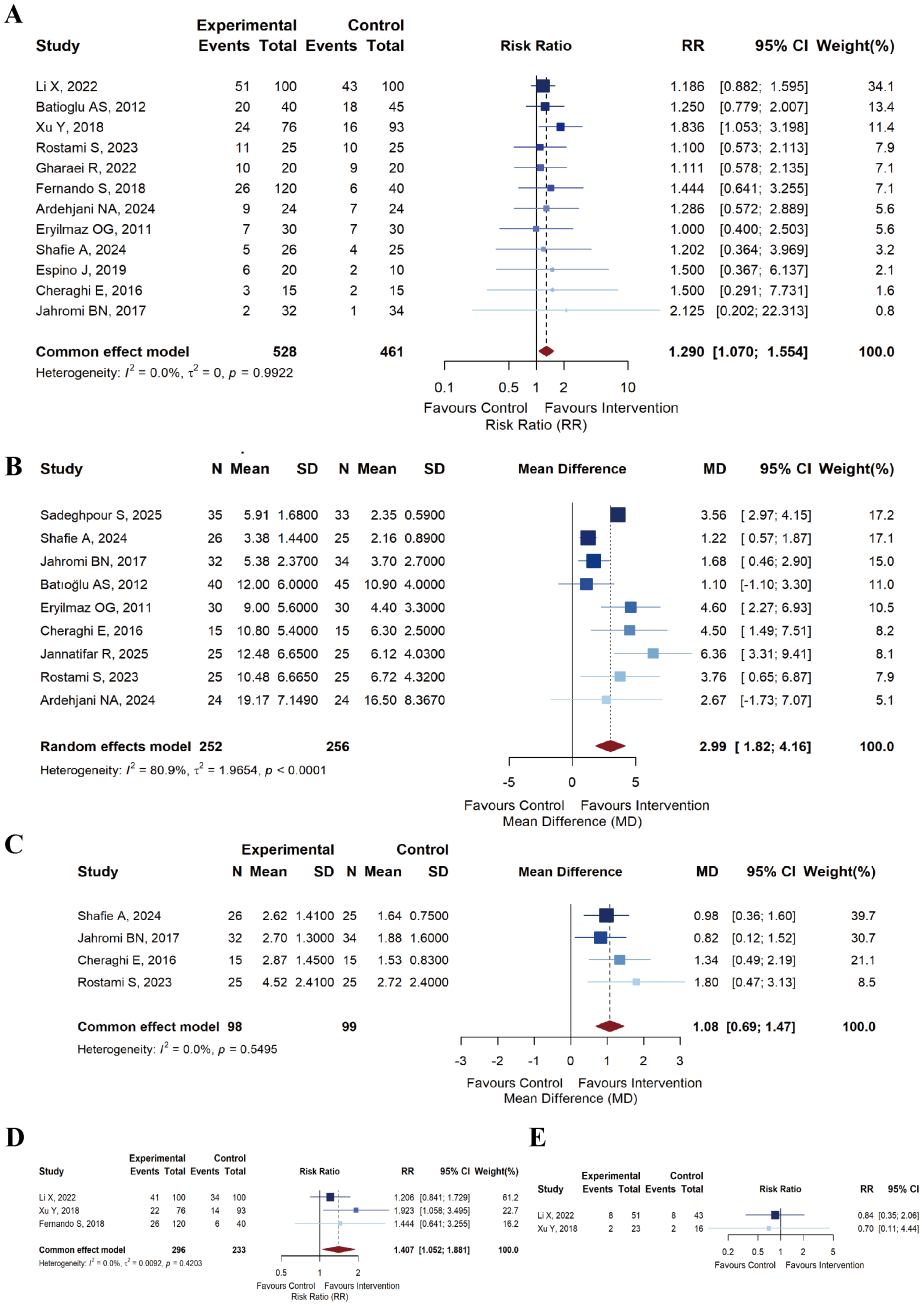
Forest plot showing individual and combined effect size estimates and 95% confidence intervals (CIs) in studies that evaluated the effect of antioxidants on outcomes of assisted reproductive technology. **(A)** clinical pregnancy rate. **(B)** the number of MII oocytes. **(C)** the number of high-quality embryos. **(D)** live birth rate. **(E)** miscarriage rate.

Among the included RCTs, 12 studies reported clinical pregnancy rates, capturing a total of 989 subjects (528 in the intervention group and 461 in the control group). Given the exceptionally low heterogeneity and highly consistent effect sizes across trials (*I*^2^ = 0%, Cochran’s Q test *P* = 0.992), a fixed-effect model was selected for the meta-analysis. The pooled results indicated that, compared with placebo or standard care, oral antioxidant supplementation significantly improved the clinical pregnancy rate [RR = 1.29 (95% CI: 1.070 - 1.554), *P* < 0.01] (**Table 3**; **Figure 3A**).

**Table 3:** Meta-analysis results.

Outcome type	Outcomes	I²	Cochran's Q test P-value	Model	Effect size	Effect value	Effect value 95 CI_lower	Effect value 95 CI_upper	Effect P-value
Primary	Clinical pregnancy rate	0%	0.9922	Common effect	Risk ratio (RR)	1.29	1.07	1.55	0.007
Primary	The number of MII oocytes	80.90%	< 0.0001	Random effect	Mean difference (MD)	2.99	1.82	4.16	< 0.001
Primary	The number of high-quality embryos	0%	0.5495	Common effect	Mean difference (MD)	1.08	0.69	1.47	< 0.001
Primary	Live birth rate	0%	0.4203	Common effect	Risk ratio (RR)	1.41	1.05	1.88	0.02
Subgroup-antioxidants type-melatonin	Clinical pregnancy rate	0%	0.9632	Random effect	Risk ratio (RR)	1.27	0.89	1.82	0.185
Subgroup-antioxidants type-N-Acetylcysteine	Clinical pregnancy rate	0%	0.7824	Random effect	Risk ratio (RR)	1.19	0.89	1.60	0.212
Subgroup-antioxidants type-astaxanthin	Clinical pregnancy rate	0%	0.9916	Random effect	Risk ratio (RR)	1.12	0.73	1.72	0.613
Subgroup-patient population-polycystic ovary syndrome	Clinical pregnancy rate	0%	0.9267	Random effect	Risk ratio (RR)	1.20	0.74	1.95	0.457
Subgroup-patient population-poor ovarian response	Clinical pregnancy rate	0%	0.5287	Random effect	Risk ratio (RR)	1.70	1.03	2.82	0.038
Subgroup-patient population-not reported	Clinical pregnancy rate	0%	0.7631	Random effect	Risk ratio (RR)	1.30	0.86	1.95	0.213
Subgroup-ovarian stimulation protocol-long protocol (GnRH agonist)	Clinical pregnancy rate	0%	0.9267	Random effect	Risk ratio (RR)	1.23	0.82	1.84	0.313
Subgroup-ovarian stimulation protocol-antagonist protocol	Clinical pregnancy rate	0%	0.9344	Random effect	Risk ratio (RR)	1.27	1.03	1.56	0.022
Subgroup-antioxidants type-melatonin	The number of MII oocytes	75.50%	0.0067	Random effect	Mean difference (MD)	2.74	1.34	4.15	< 0.001
Subgroup-antioxidants type-astaxanthin	The number of MII oocytes	59.20%	0.1176	Random effect	Mean difference (MD)	2.02	-0.29	4.32	0.0871
Subgroup-patient population-diminished ovarian reserve	The number of MII oocytes	86.40%	0.0067	Random effect	Mean difference (MD)	2.70	0.86	4.54	0.0055
Subgroup-patient population-polycystic ovary syndrome	The number of MII oocytes	0%	0.5013	Random effect	Mean difference (MD)	3.92	1.43	6.40	0.002
Subgroup-patient population-endometriosis	The number of MII oocytes	26.90%	0.2422	Random effect	Mean difference (MD)	5.08	2.53	7.63	< 0.001
Subgroup-ovarian stimulation protocol-long protocol (GnRH agonist)	The number of MII oocytes	69.10%	0.0114	Random effect	Mean difference (MD)	2.95	1.68	4.21	< 0.001
Subgroup-ovarian stimulation protocol-antagonist protocol	The number of MII oocytes	76.40%	0.0053	Random effect	Mean difference (MD)	3.31	0.67	5.96	0.0141

#### 3.4.2 MII oocytes

Nine studies evaluated the number of MII oocytes, comprising 252 subjects in the intervention group and 256 in the control group. To account for the high heterogeneity and marked variations across individual trials (*I*^2^ = 80.9%, Cochran’s Q test *P* < 0.0001), a random-effects model was employed. The pooled estimates revealed that antioxidant therapy significantly increased the yield of MII oocytes retrieved from women undergoing IVF/ICSI [MD = 2.99 (95% CI: 1.82 – 4.16), *P* < 0.001]. Detailed findings are presented in **Table 3**, and the forest plot is displayed in **Figure 3B**.

#### 3.4.3 High-quality embryo

The acquisition of high-quality embryos represents a pivotal stage in ART success. Of the 14 included trials, 4 trials provided details on the number of high-quality embryos, tracking 98 patients in the intervention group and 99 in the control group. Heterogeneity testing revealed negligible variance across studies (*I*^2^ = 0%, Cochran’s Q test *P* = 0.5495), justifying the use of a fixed-effect model. The pooled effect size verified that antioxidant supplementation exerts a highly significant, positive impact on increasing the number of high-quality embryos retrieved [MD = 1.08 (95%CI: 0.69–1.47), *P* < 0.001] (**Table 3**; **Figure 3C**).

#### 3.4.4 Live birth

Only 3 studies reported live birth rates. The intra-study heterogeneity was remarkably low (*I*^2^ = 0%, Cochran’s Q test *P* = 0.4203). The fixed-effect meta-analysis revealed that oral antioxidants achieved a significant improvement in the live birth rate following IVF/ICSI-ET [RR = 1.407 (95% CI: 1.052 - 1.881), *P* < 0.05] (**Table 3**; **Figure 3D**).

#### 3.4.5 Miscarriage

Because only 2 trials reported miscarriage rates, a quantitative synthesis of effect sizes was precluded due to the limited number of studies. Nevertheless, a qualitative review indicated that both trials showed a downward trend in miscarriage risk within the antioxidant group, as evidenced by point estimates below 1.0, though their 95% CIs crossed the unity (1.0), indicating a statistically non-significant difference. Specifically, Li X et al. (2022) (16) observed a miscarriage rate of 15.7% (8/51) in the antioxidant group versus 18.6% (8/43) in the control group, RR = 0.84 (95% CI: 0.35 – 2.06); Similarly, Xu Y et al. (2018) (13) reported a miscarriage rate of 8.7% (2/23) under antioxidant treatment compared with 12.5% (2/16) in the control group [RR = 0.70 (95% CI: 0.11 – 4.44)]. **Figure 3E** presents a detailed breakdown of the miscarriage rate findings from these two studies.

### 3.5 Exploratory subgroup analysis

To further dissect the clinical impacts of distinct antioxidant formulations, COS protocols, and underlying etiologies, we conducted stratified analysis on two major outcomes—clinical pregnancy rate and the number of MII oocytes—contingent upon adequate study volume within subsets. Of note, the following subgroup findings are based on a limited number of studies and should be considered exploratory.

**Figure 4:**
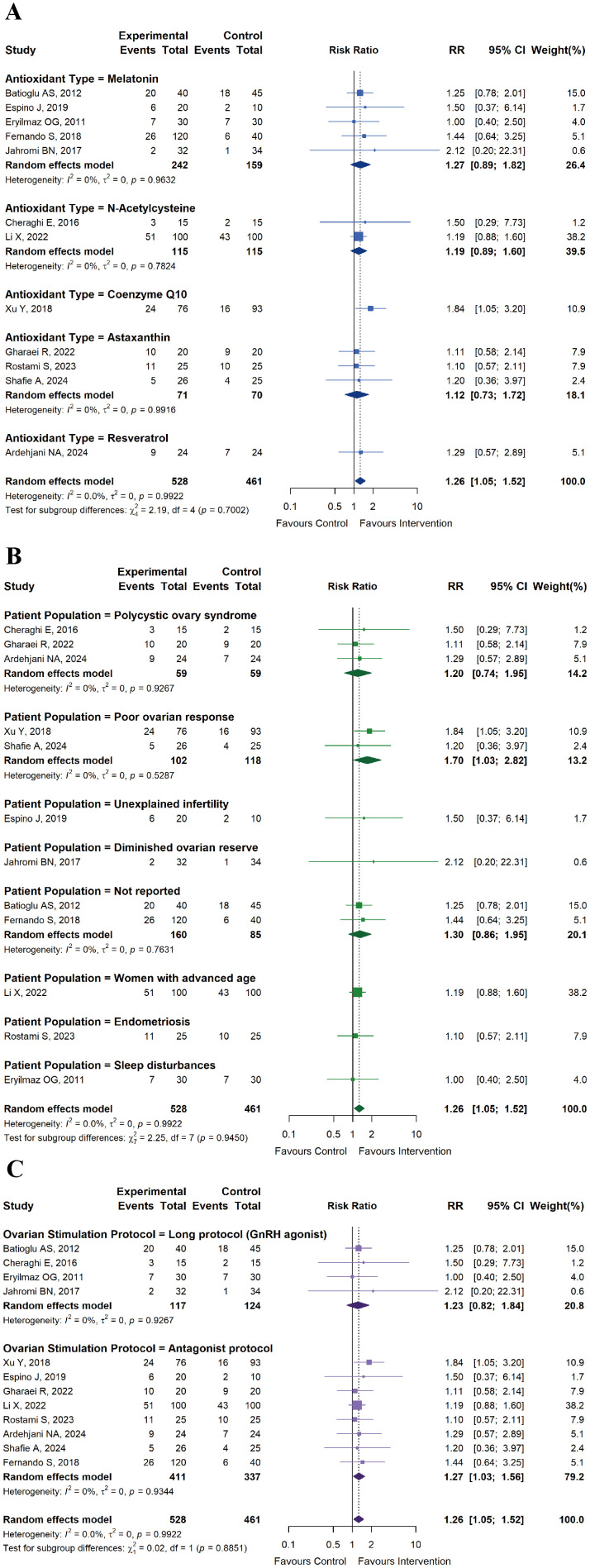
Subgroup analysis of the effect of antioxidants on clinical pregnancy rate of assisted reproductive technology. **(A)** different antioxidant types. **(B)** different patient population. **(C)** different ovarian stimulation protocol.

For the clinical pregnancy rate, stratification by antioxidant type revealed negligible intra-group heterogeneity, reflecting highly uniform effects within identical drug classes. Although the pooled RRs for melatonin, N-acetylcysteine, and astaxanthin clustered within a promising range of 1.12 to 1.27, their respective 95% CIs all crossed 1.0, failing to achieve individual statistical significance. This suggests that while these three oral antioxidants may confer potential clinical trends toward improving clinical pregnancy rates, definitive confirmation is still required (**Figure 4A**; **Table 3**). When stratified by baseline pathology, data pooling was feasible only for PCOS, POR, and “not reported” cohorts. These sub-analyses demonstrated very low intra-group heterogeneity with RRs ranging from 1.20 to 1.70; however, most 95% CIs spanned across 1.0, with the notable exception of the POR subgroup. This indicates that oral antioxidant supplementation yields a highly probable and significant clinical benefit specifically for the POR population [RR = 1.70 (95% CI: 1.03 – 2.82), *P* < 0.05] (**Figure 4B**; **Table 3**). Regarding COS strategies, both the long protocol (4 trials) and the antagonist protocol (8 trials) satisfied the minimum sample size criteria for meta-analytic synthesis. The subgroup analysis demonstrated extremely low intra-group heterogeneity. The pooled effect size for the long protocol group was RR = 1.23 [(95% CI: 0.82 – 1.84), *P* = 0.3127], given that its 95% CI encompassed 1.0 and did not reach statistical significance, it implies only a potential clinical trend. Conversely, the antagonist protocol subgroup yielded a pooled RR of 1.27 [(95% CI: 1.03 – 1.56), *P* = 0.0224 < 0.05], demonstrating a statistically significant improvement. This highlights that oral antioxidant supplementation provides a significant clinical advantage in elevating clinical pregnancy rates specifically for patients undergoing IVF/ICSI-ET managed with the antagonist protocol (**Figure 4C**; **Table 3**).

**Figure 5:**
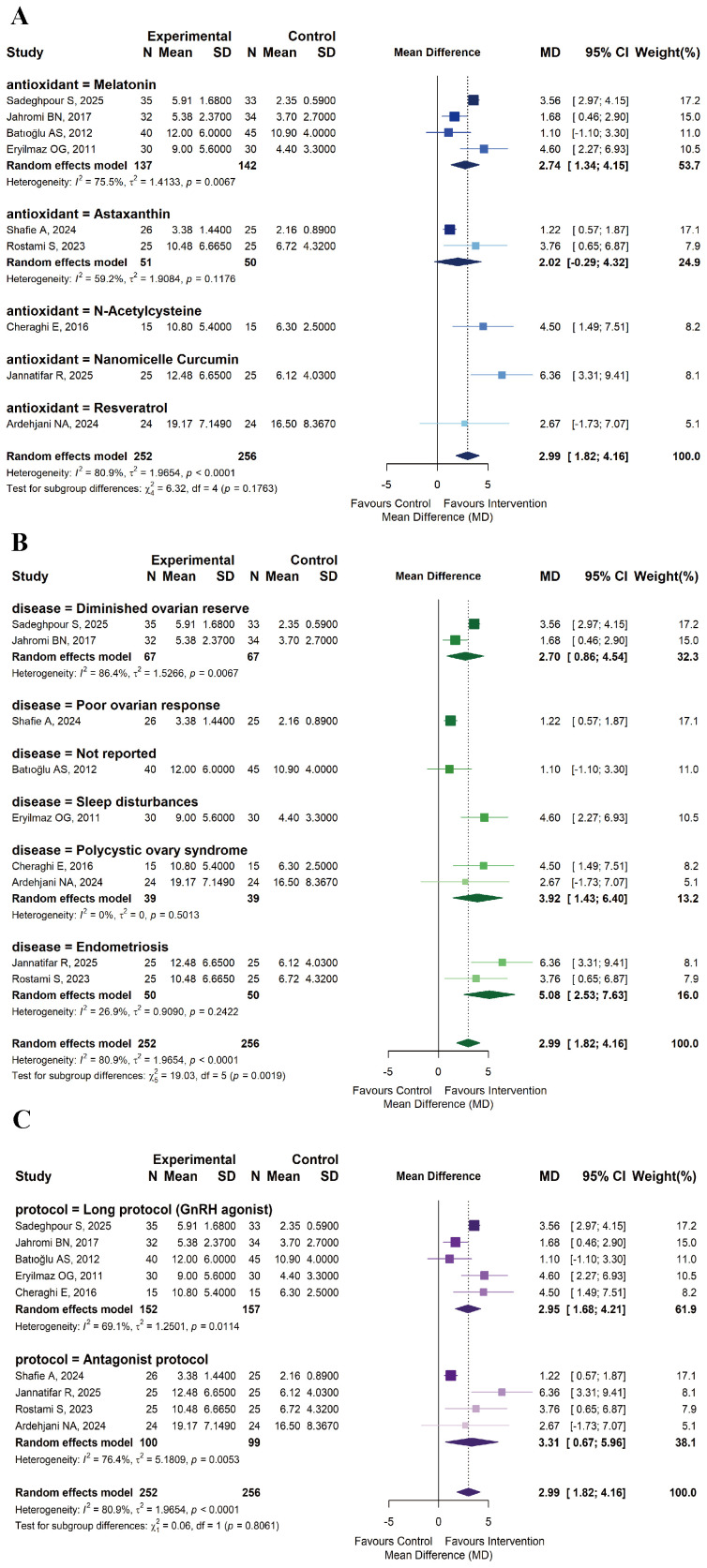
Subgroup analysis of the effect of antioxidants on the number of MII oocytes in assisted reproductive technology. **(A)** different antioxidant types. **(B)** different patient population. **(C)** different ovarian stimulation protocol.

Given that oocyte yield is an indispensable factor dictating ART success, we carefully examined the number of MII oocytes. In the subgroup analysis based on antioxidant categories, only melatonin and astaxanthin possessed a sufficient number of trials to permit pooling. Astaxanthin yielded an MD of 2.02 (95% CI: -0.29 – 4.32, *P* = 0.0871), which despite hinting at a clinical benefit, remains inconclusive as its CI crossed zero. In contrast, the melatonin subgroup demonstrated a robust and significant increase in mature oocyte count [MD = 2.74 (95% CI: 1.34 – 4.15), *P* < 0.001], validating that oral melatonin intake is highly effective in increasing the number of MII oocytes during IVF/ICSI cycles (**Figure 5A**; **Table 3**). To investigate the influence of baseline maternal conditions, etiology-based sub-analyses were undertaken for PCOS, endometriosis, and DOR cohorts, all of which met the baseline inclusion threshold. The PCOS subgroup displayed remarkably low intra-group heterogeneity, culminating in a significant pooled increase MD = 3.92 (95% CI: 1.43 – 6.40), *P* < 0.05. The endometriosis subgroup similarly showed minimal heterogeneity and a pronounced improvement [MD = 5.08 (95% CI: 2.53 – 7.63), *P* < 0.001]. However, the DOR subgroup exhibits substantial heterogeneity, with large variations among the included studies [MD = 2.70 (95% CI: 0.86 – 4.54), *P* < 0.05]. Taken together, the pooled MDs for PCOS, endometriosis, and DOR were consistently above zero with entirely positive CIs and high statistical significance, suggesting that oral antioxidant supplementation is associated with improved outcomes in MII oocyte retrieval across these three patient populations (**Figure 5B**; **Table 3**). Finally, stratification by COS regimens revealed elevated intra-group heterogeneity in both subsets; nonetheless, oral antioxidants significantly boosted the number of retrieved MII oocytes in both the long protocol [MD = 2.95 (95% CI: 1.68 – 4.21), *P* < 0.001] and the antagonist protocol [MD = 3.31 (95% CI: 0.67 – 5.96), *P* < 0.05], reinforcing its broad clinical utility across different ovarian stimulation regimens (**Figure 5C**; **Table 3**).

### 3.6 Sensitivity analysis

**Figure 6:**
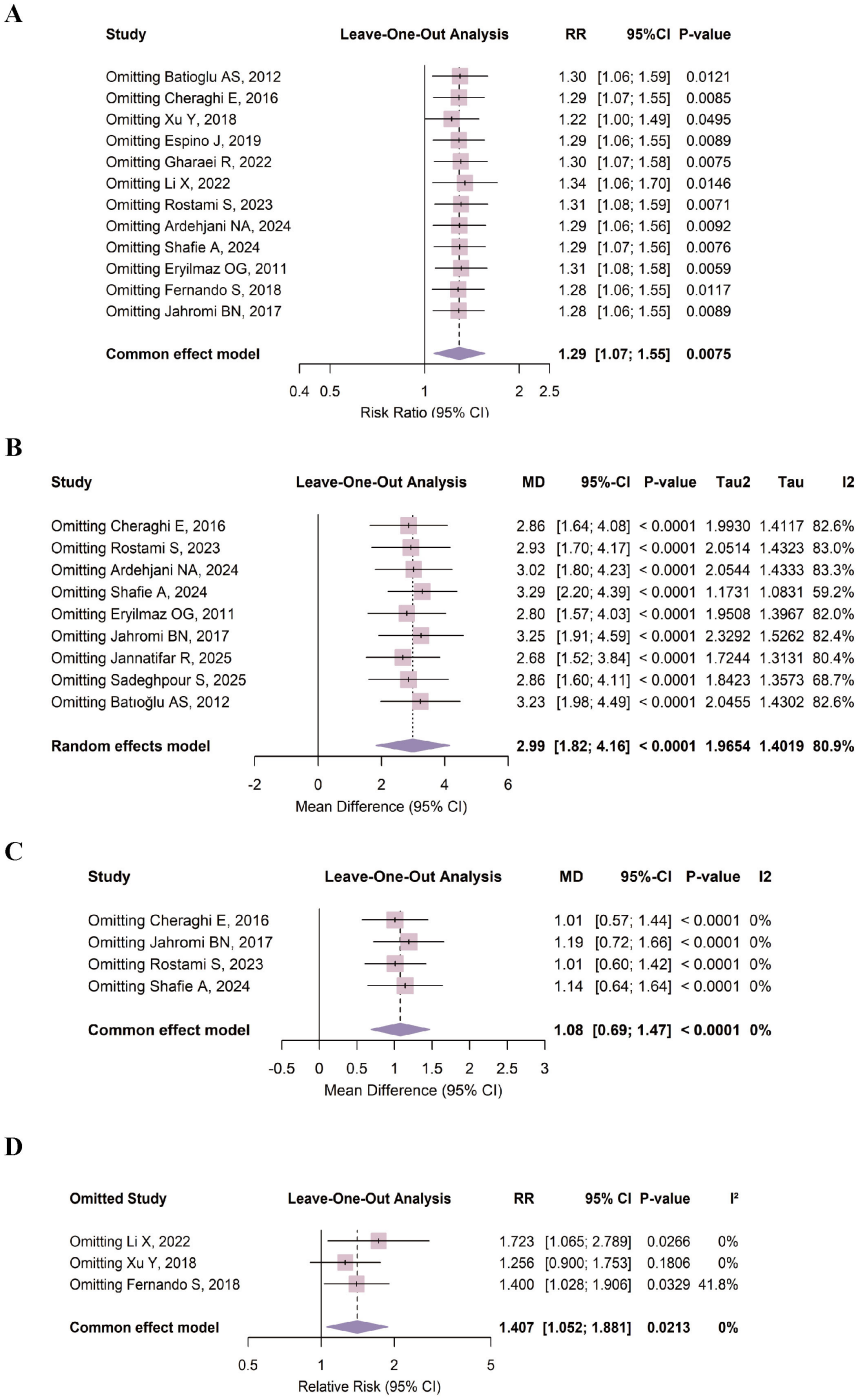
Leave-one-out sensitivity analysis plot. **(A)** clinical pregnancy rate. **(B)** the number of MII oocytes. **(C)** the number of high-quality embryos. **(D)** live birth rate.

To verify the stability and robustness of our combined estimates, a leave-one-out sensitivity analysis was performed. For the clinical pregnancy rate, iterative removal of any single trial under the fixed-effect model yielded pooled RRs consistently bounded between 1.22 and 1.34, with 95% CIs firmly tracking between 1.00 and 1.70, preserving a stable significance level of *P* < 0.05. Crucially, all individual point estimates fell well within the 95% CI of the overall primary analysis, and no directional reversals occurred (all RRs remained > 1.0). This demonstrates that the synthesized clinical pregnancy rate outcome is highly robust and free from undue distortion caused by any single influential study (**Figure 6A**). For the number of MII oocytes, the leave-one-out analysis under the random-effects model showed that the pooled MD remained consistent, ranging from 2.68 to 3.29. All 95% CIs excluded zero (lower bounds: 1.52 - 2.20), confirming the robustness of the oocyte data against the influence of any single study (**Figure 6B**). Regarding the number of high-quality embryos, sensitivity analysis demonstrated that the MD fluctuated between 1.01 and 1.19, with all estimates remaining statistically significant (**Figure 6C**). Regarding the live birth rate, while a consistent trend toward clinical benefit was observed across all iterations (RR ranging from 1.256 to 1.723; all RR > 1.0), the result lost statistical significance after the exclusion of Xu Y et al. (2018) (13), with the 95% CI crossing 1.0 (95% CI: 0.90–1.753). This shift suggests that the pooled estimate for live birth rate is notably sensitive to this specific study, likely due to the limited number of included trials; consequently, this finding should be interpreted with caution (**Figure 6D**). Finally, sensitivity analyses were omitted for the miscarriage rate due to the scarcity of data (fewer than 3 studies), necessitating a conservative interpretation of this outcome.

**Figure 7:**
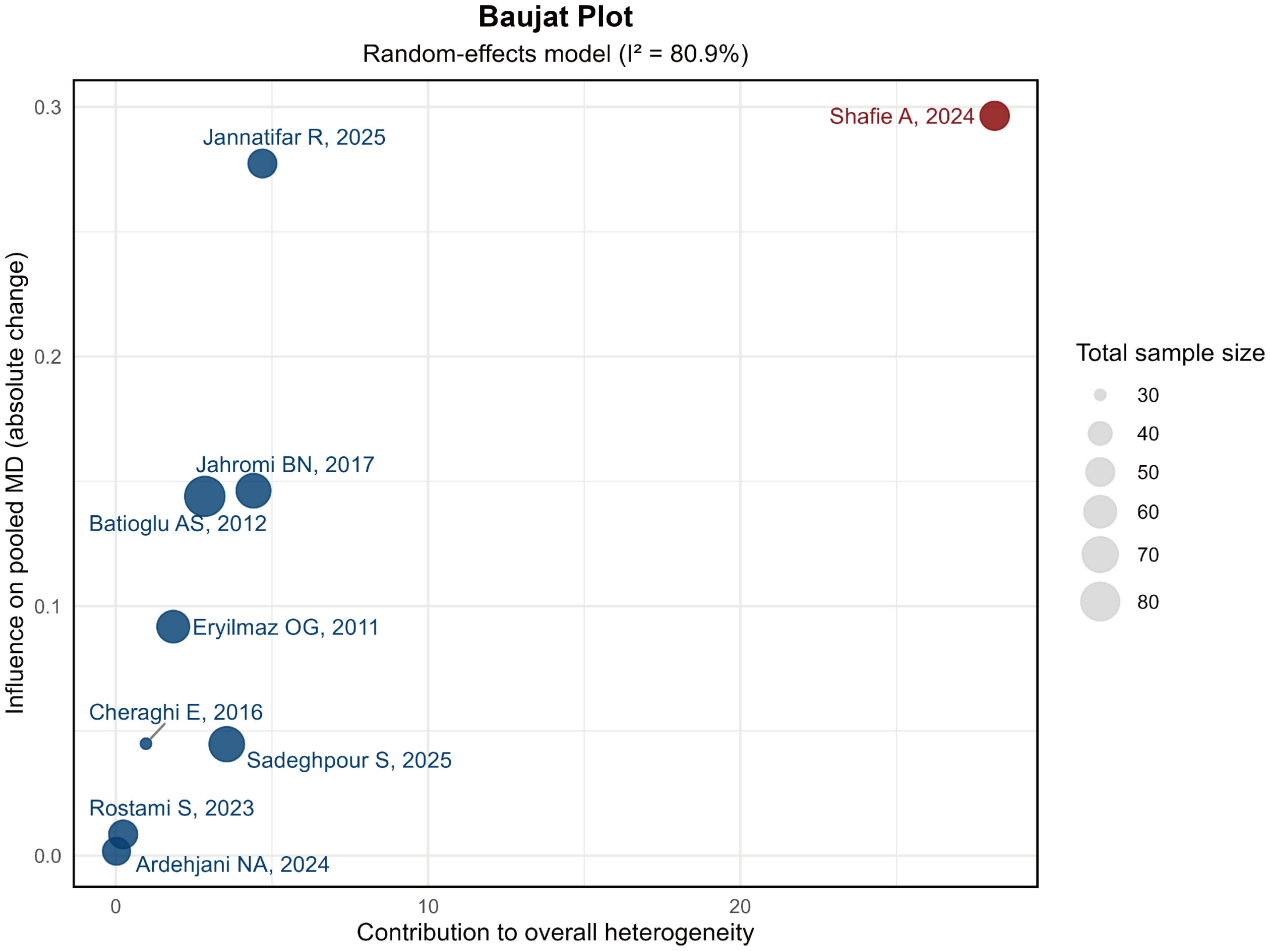
Baujat plot of the number of MII oocytes.

To further explore potential sources of the high heterogeneity observed in the number of MII oocytes, a Baujat plot analysis was conducted. The results indicated that the study by Shafie A et al. (2024) (19) was prominently located in the upper right quadrant of the plot, contributing disproportionately more to both the overall heterogeneity and the pooled effect size than the other studies, suggesting that it is a key study influencing the overall heterogeneity and the combined results (**Figure 7**). The leave-one-out analysis confirmed that after removing Shafie A et al. (2024) (19), the pooled MD increased from [2.99 (95% CI: 1.82 – 4.16), *P* < 0.001] to [3.29 (95% CI: 2.20 – 4.39), *P* < 0.001], while the *I*^2^ decreased from 80.9% to 59.2% (**Figure 6B**), representing the largest reduction observed across all leave-one-out iterations. To investigate whether age exerted a potential moderating effect on the number of MII oocytes, we performed a univariate meta-regression analysis with age as a covariate. The results did not reveal a statistically significant association between age and the pooled effect size (coefficient = -0.2622, 95% CI: -0.5844 - 0.06, *P* = 0.111). However, the relatively small number of included studies (n = 9) may limit the statistical power to identify such associations or other potential sources of heterogeneity.

### 3.7 Publication bias analysis

**Figure 8:**
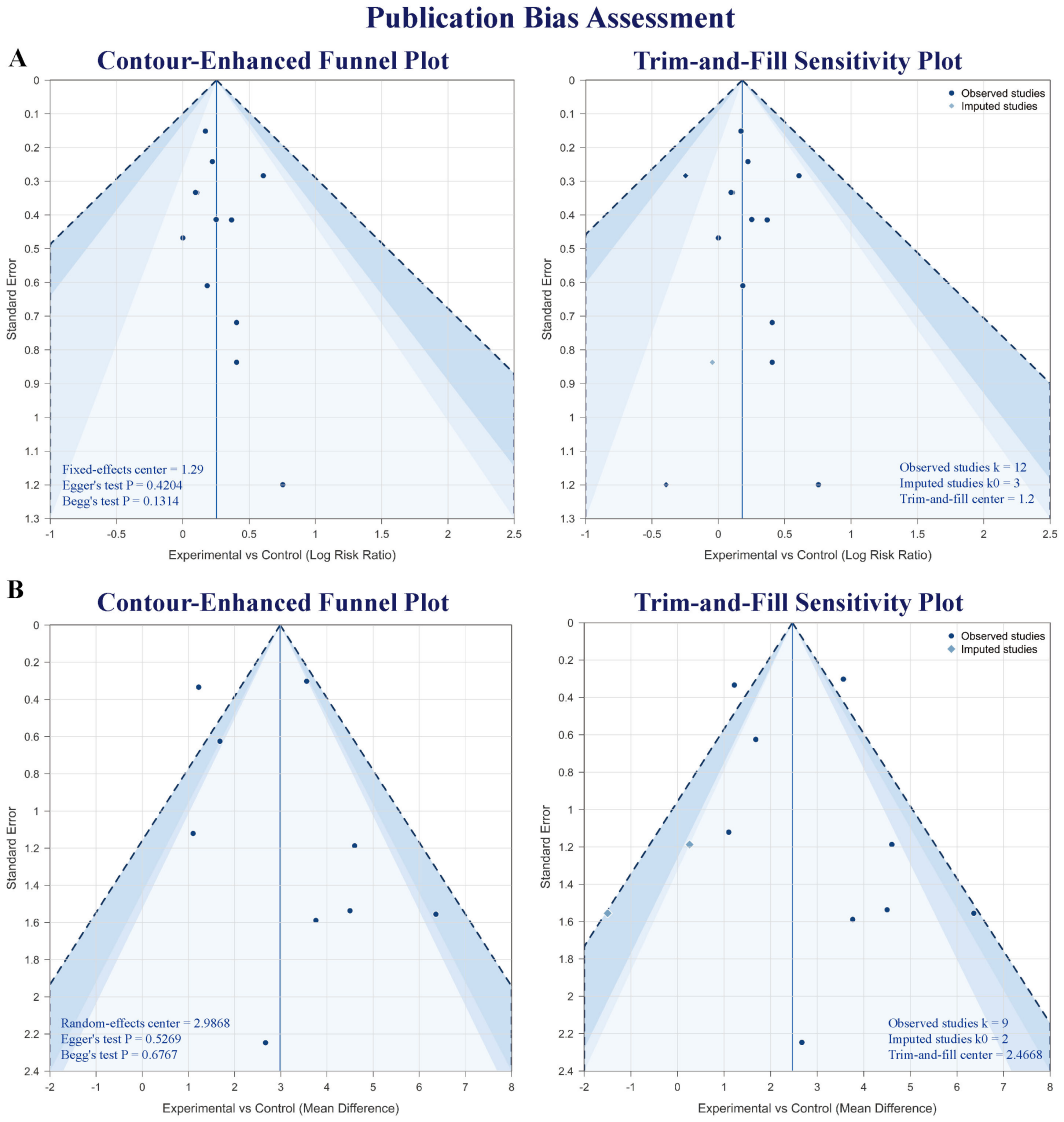
Publication bias analysis of the meta-analysis. **(A)** clinical pregnancy rate. **(B)** the number of MII oocytes.

Potential publication bias was rigorously screened using funnel plots alongside Egger’s linear regression and Begg’s rank correlation tests, followed by the trim-and-fill correction method where appropriate. However, the reliability of publication bias assessments is limited when the number of included studies is small. Consequently, we did not perform publication bias analyses for the number of high-quality embryos, live birth rate, and miscarriage rate due to the insufficient number of available trials. For the clinical pregnancy rate, the funnel plot exhibited a mild asymmetry. However, Egger’s test (*P* = 0.4204) and Begg’s test (*P* = 0.1314) both exceeded 0.05. Application of the trim-and-fill method imputed 3 theoretically missing studies, adjusting the total trial count from 12 to 15. The adjusted pooled effect size (RR = 1.20) was slightly attenuated compared with the original estimate (RR = 1.29), suggesting potential small-study bias. The adjusted effect estimate remained above 1.0 (RR = 1.20, 95%CI: 1.01 – 1.42), and the direction of benefit favoring the antioxidant group was unchanged. Taken together, while the trim-and-fill adjustment suggests the possibility of a modest small-study bias that may have slightly overestimated the effect magnitude, the overall evidence remains robust, and the primary conclusion of a beneficial effect of antioxidants on clinical pregnancy rate is not materially altered by this potential bias (**Figure 8A**). For the number of MII oocytes, the funnel plot was structurally symmetrical. Both Egger’s (*P* = 0.5269) and Begg’s (*P* = 0.6767) tests were performed, neither of which suggested significant funnel plot asymmetry. In addition, the trim-and-fill method estimated 2 potentially missing studies, increasing the total from 9 to 11. After imputing these studies, the adjusted pooled MD remained 2.4668, with a direction of effect consistent with the original estimate, supporting the robustness of our findings (**Figure 8B**). Overall, despite mild funnel plot asymmetry and possible small-study effects, no statistically significant publication bias was detected for either outcome, and the direction of the adjusted effect estimates remained unchanged, supporting the robustness of the findings.

### 3.8 Evidence certainty

The GRADE assessment indicated that the overall evidence generated by this meta-analysis is robust. Specifically, the evidence certainty for the clinical pregnancy rate was rated as “high, “ as no downgrading was required in the assessment. For the number of MII oocytes, although high heterogeneity was observed among the included studies (*I*^2^ = 80.9%), leave-one-out sensitivity analyses confirmed that no single study dominated the pooled effect (MD range: 2.68–3.29, all *P* < 0.0001). Therefore, the certainty of evidence for this outcome was downgraded by one level due to inconsistency, resulting in a “moderate” rating. Regarding the number of high-quality embryos and live birth rates, the evidence was downgraded by one level for publication bias under the “other considerations” domain, given the limited number of studies, the lack of large-scale multicenter RCTs, and the inability to assess publication bias; thus, the final GRADE certainty was also determined to be “moderate” (**Table 4**). In summary, all four primary outcomes supporting our meta-analysis demonstrated acceptable levels of evidence certainty. Nevertheless, given the limited number of included studies, we remain conservative in our assessment of the certainty of these findings.

**Table 4:** GRADE assessment and evidence profile.

Certainty assessment							No of patients		Effect		Certainty	Importance
No of studies	Study design	Risk of bias	Inconsistency	Indirectness	Imprecision	Other considerations	Oral antioxidant	Placebo or standard care	Relative (95% CI)	Absolute (95% CI)		
Clinical pregnancy rates												
12	randomised trials	not serious	not serious	not serious	not serious	none	528/989 (53.4%)	461/989 (46.6%)	RR 1.29 (1.07 to 1.55)	135 more per 1, 000 (from 33 more to 258 more)	⨁⨁⨁⨁ High	CRITICAL
The number of MII oocytes												
9	randomised trials	not serious	seriousa	not serious	not serious	none	252	257	–	MD 2.99 more (1.82 more to 4.16 more)	⨁⨁⨁◯ Moderate^a^	CRITICAL
The number of high-quality embryos												
4	randomised trials	not serious	not serious	not serious	not serious	publication bias strongly suspectedb	98	99	–	MD 1.18 more (0.76 more to 1.6 more)	⨁⨁⨁◯ Moderate^b^	CRITICAL
Live birth rates												
3	randomised trials	not serious	not serious	not serious	not serious	publication bias strongly suspectedb	296/529 (56.0%)	233/529 (44.0%)	RR 1.41 (1.05 to 1.88)	179 more per 1, 000 (from 23 more to 388 more)	⨁⨁⨁◯ Moderateb	CRITICAL

CI, confidence interval; MD, mean difference; RR, risk ratio.

Question: The effects of oral antioxidants on outcomes of assisted reproductive technology: a systematic review and meta-analysis of randomized controlled trials.

Explanations:

a. Serious (−1): Downgraded one level for inconsistency (I² = 80.9%) and sensitivity (leave-one-out) analyses confirmed no single study dominated the pooled effect.

b. Serious (−1): Formal publication bias assessment (e.g., funnel plot) not feasible due to insufficient studies.

## 4 Discussion

Infertility is a disease defined as the failure to achieve a clinical pregnancy, representing a major global health challenge and a significant contributor to substantial economic burden (1, 25, 26). The remarkable progress and sustained application of ART have provided crucial technical support in addressing fertility issues for patients (2, 27). Due to factors such as oocyte and embryo quality, and female age, reproductive outcomes of ART remain suboptimal and the available evidence is not yet definitive, necessitating careful clinical decision-making between therapeutic efficacy and potential risks (27). Enhancing the success rate of ART procedures remains a core issue in the reproductive field. OS has been identified as one of the critical mechanisms contributing to diminished oocyte and embryo quality, which is closely linked to female reproductive disorders (5). In recent years, antioxidant therapy has shown promising potential as an adjuvant strategy for improving oocyte quality, increasing the yield of high-quality embryos, reducing aneuploidy incidence, and enhancing ART success rates (6, 28).

To evaluate the overall trend of oral antioxidant supplementation as an adjuvant therapy on reproductive outcomes in IVF/ICSI-ET cycles and to explore the potential therapeutic effects of mitigating OS, we conducted this systematic review and meta-analysis. Given our objective to assess the broad trend of oral antioxidants, the included trials comprised a variety of agents (melatonin, N-acetylcysteine, coenzyme Q10, astaxanthin, resveratrol, and nanomicelle curcumin), which naturally differed in dosage, duration, timing relative to COS, and patient characteristics. Such diversity inevitably introduced clinical and methodological heterogeneity, which may influence the pooled effect estimates. Although we employed rigorous methods—including heterogeneity testing, selection of appropriate effect models, and exploration of heterogeneity sources for high-heterogeneity outcomes—the findings should be interpreted with caution due to the limited number of included studies. Notably, the high heterogeneity observed in the number of MII oocytes appears more sensitive to these clinical variations; while sensitivity analysis indicated that this heterogeneity was not driven by any single study, the results warrant cautious interpretation. Conversely, no statistically significant heterogeneity was detected for clinical pregnancy rate, number of high-quality embryos, or live birth rate, suggesting that these findings are relatively robust, although limitations arising from the small sample size persist. Consequently, our analysis reveals an overall trend in the therapeutic potential of antioxidants as an ART adjuvant; however, future high-quality evidence utilizing standardized antioxidant regimens is essential to substantiate these findings and guide clinical decision-making.

Mature oocytes, high-quality embryos, clinical pregnancy, and live birth represent key, temporally linked stages of an ART cycle. Our meta-analysis indicates that oral antioxidant supplementation during IVF/ICSI-ET cycles exhibits a consistent trend toward clinical benefit across these endpoints. Although the miscarriage rate analysis included only two studies, both suggest a potential reduction in miscarriage risk with antioxidant use. Collectively, our results suggest that oral antioxidant adjuvant therapy may positively influence ART outcomes through multiple mechanisms. Nevertheless, given the limitations in study volume, subgroup findings should be considered exploratory rather than definitive.

While low levels of ROS are indispensable for physiological cellular signaling and regular embryonic development, excessive ROS generation inevitably precipitates OS. This pathobiological state acts as a key executioner of compromised oocyte quality and disrupted endometrial receptivity, while simultaneously fracturing embryonic development by driving DNA fragmentation and chromosomal aneuploidy (5, 29, 30). Follicular fluid harvested from patients with PCOS, endometriosis, or advanced reproductive age frequently presents a deeply exaggerated OS profile (5). Concurrently, standard ART procedures—including controlled ovarian stimulation, prolonged *in vitro* incubation of gametes and embryos, and artificial fertilization techniques—inherently expose biological tissues to non-physiological environments, which drastically accelerates ROS accumulation, depletes baseline antioxidant reserves, and provokes severe iatrogenic OS, ultimately compounding the risk of adverse reproductive outcomes (30, 31). Antioxidant administration stands out as a core strategy to re-establish redox equilibrium. Although our pooled results suggest that oral antioxidants may offer potential clinical benefits in improving the clinical pregnancy rate, MII oocyte count, high-quality embryo yield, and live birth rate, the data for high-quality embryos and live birth rates were derived from fewer than five trials, demanding a conservative interpretation. Additionally, a high level of heterogeneity was noted during the continuous data synthesis for MII oocytes. Consequently, the clinical extrapolation of antioxidant-mediated improvements in MII oocyte yield requires diligent scrutiny.

Regarding the number of MII oocytes, our meta-analysis indicates a statistically significant beneficial effect of antioxidant supplementation, suggesting potential clinical utility. However, significant heterogeneity was observed (*I*^2^ = 80.9%). Although sensitivity analyses demonstrated that the pooled MD fluctuated strictly between 2.68 and 3.29 (all *P* < 0.0001), regardless of the individual study excluded—with all 95% CIs being positive and statistically significant—the high level of heterogeneity limits the robustness of our findings. Further large-scale RCTs with standardized antioxidant protocols are warranted to derive more definitive conclusions.

In exploring the sources of heterogeneity for the number of MII oocytes, sensitivity analysis revealed that excluding the study by Shafie A et al. (2024) (19) significantly reduced heterogeneity to 59.2%, a finding corroborated by the Baujat plot. This study was a randomized, triple-blind, placebo-controlled trial; however, it differed from other included studies as the only one focusing on patients with POR, who represent the category with the poorest prognosis. A hallmark of POR is a low yield of retrieved oocytes and a suboptimal response to COS. Given that this outcome is highly dependent on ovarian function, and despite the fact that the underlying mechanism of POR is closely linked to OS and the antioxidant used was astaxanthin, the therapeutic potential of antioxidants in POR patients may be constrained by the severity of poor ovarian response itself, potentially leading to an underestimation of the true efficacy (19). Sensitivity analysis supported this: after excluding this study, the pooled MD increased from 2.99 (95% CI: 1.82–4.16) to 3.29 (95% CI: 2.20–4.39), which represents the largest shift among all leave-one-out iterations. Furthermore, subgroup analyses by population characteristics provided corroborating evidence: lower heterogeneity was observed in the PCOS (*I*^2^ = 0%) and Endometriosis (*I*^2^ = 26.9%) subgroups, whereas the DOR subgroup exhibited extremely high heterogeneity (*I*^2^ = 86.4%). Both studies in the DOR subgroup utilized 3 mg of melatonin and a long protocol for COS; beyond the 8-year gap in publication dates, the primary discrepancy lay in the baseline ovarian function. Specifically, in the treatment group, the study by Jahromi BN et al. (2017) reported an AMH of 0.98 ± 0.63 and an AFC of 6.1 ± 3.7, whereas Sadeghpour S et al. (2025) reported lower values (AMH 0.79 ± 0.17; AFC 4.24 ± 1.23), indicating more severe diminished ovarian reserve in the latter (21, 24). These findings suggest that variations in ovarian reserve are associated with—and likely contribute to—the observed heterogeneity. However, given the limited number of studies in each subgroup (n=2), the robustness of these findings is insufficient, and they should be interpreted as exploratory.

High-quality embryos are critical to ART success. After retrieving a sufficient number of MII oocytes, a complex process of fertilization, cleavage, and subsequent development is required to form a high-quality embryo with developmental potential. Our meta-analysis indicates that antioxidant adjuvant therapy shows promising potential in increasing the number of high-quality embryos. Despite the minimal heterogeneity and robust sensitivity analysis, this finding is based on only four trials and requires further verification. The observed benefits in MII oocytes and high-quality embryos suggest that the latter may be a downstream improvement mediated by the former, though this hypothesis warrants further exploration.

Our findings suggest that oral antioxidant supplementation during IVF/ICSI-ET cycles may improve clinical benefits for clinical pregnancy rates, with robust results in our meta-analysis. Although the funnel plot suggests potential small-study effects, this metric exhibits higher reliability compared to others in our analysis. Clinical pregnancy is a pivotal milestone in the ART treatment pathway, following oocyte maturation and embryo development, and relies on successful implantation and the establishment of early pregnancy. We speculate that the benefit to clinical pregnancy rates may be a cumulative result of improved oocyte maturity and embryo quality, though the specific associations remain to be elucidated. Exploratory subgroup analysis suggests that while oral antioxidant supplementation significantly boosts MII oocyte yield uniformly across both long GnRH agonist and antagonist protocols, its capacity to significantly improve the clinical pregnancy rate was observed only in the antagonist protocol. This observation should be considered exploratory and may relate to the differences in OS risk and endometrial receptivity profiles associated with these two COS protocols.

For live birth and miscarriage rates, although our meta-analysis indicates a potential positive trend for antioxidant supplementation, the limited number of included studies precludes definitive conclusions. These represent the most crucial outcomes in reproductive medicine, serving as key references for clinical guidance. There is an urgent need for large-scale, multicenter RCTs that utilize standardized antioxidant regimens and adopt live birth and miscarriage as primary endpoints.

Among the different types of antioxidants, melatonin showed more consistent potential benefits in increasing mature oocytes and improving clinical pregnancy. This resonates with a prior meta-analysis which noted that melatonin supplementation exerts favorable actions on oocyte and embryo architecture, particularly in women facing PCOS or DOR (30). Melatonin is an indoleamine hormone secreted by the pineal gland of the central nervous system under dark conditions, that is produced through a series of enzymatic reactions (32, 33). Melatonin regulates OS, immune response, inflammation, and microenvironmental angiogenesis by upregulating the gene expression of various antioxidant enzymes and reducing ROS levels, thereby improving oocyte quality and endometrial receptivity (34). Findings from previous studies have also demonstrated that the concentration of melatonin is highly likely to preserve ovarian reserve and boost IVF outcomes in advanced maternal age cohorts (specifically those ≥ 38 years old) by preserving mitochondrial integrity and reprogramming energy metabolism via its potent antioxidant properties (35). Although several meta-analyses have previously evaluated melatonin in ART, a rigorous, RCT-exclusive synthesis capable of providing highly granular clinical stratification remains scarce. This gap strongly reinforces the urgency for future large-scale, multi-center, high-quality RCTs to consolidate evidence-based guidelines for clinical practice.

Different infertility etiologies are driven by distinct pathophysiological mechanisms, in which the role and intensity of OS vary; consequently, the efficacy of antioxidant supplementation may also differ. Oxidative damage is a primary pathophysiological mechanism in the metabolic and reproductive abnormalities of PCOS (36). Our exploratory subgroup analysis aligns with this finding, showing consistent therapeutic effects in the PCOS subgroup (MII oocytes: MD = 3.92; clinical pregnancy: RR = 1.20) with extremely low heterogeneity (*I²* = 0%). Conversely, POR and DOR are characterized by progressive depletion of ovarian reserve and diminished oocyte quality; while OS may be a contributing factor, it is likely not the dominant mechanism (37). These factors, combined with diagnostic heterogeneity, significantly influence the variation in antioxidant efficacy and explain the high heterogeneity in MII oocyte counts. Therefore, our pooled results provide an average estimate of the treatment effect across diverse populations and should not be directly extrapolated to specific subgroups. Clinical decision-making must be individualized based on patient age, etiology, and ovarian reserve. Antioxidants should be targeted rather than used as a universal adjuvant for all ART patients. Future high-quality, large-scale RCTs are necessary to establish definitive clinical guidelines, and periodic updates to this meta-analysis will remain of high clinical value.

## 5 Limitations

Several limitations must be taken into account when interpreting the findings of this meta-analysis. First, although the aggregate number of included trials was statistically sufficient for data pooling, the total sample size remains somewhat modest for drawing definitive, high-impact clinical conclusions, especially for the number of high-quality embryos and the live birth rate. Second, due to the limited number of eligible trials, a quantitative meta-analysis assessing the impact of antioxidants on the miscarriage rate could not be performed; this highlights the need for future large-scale, multi-center RCTs to conclusively determine if oral antioxidants serve as an independent protective factor against miscarriage. Third, the clinical diversity inherent in the included studies—characterized by varying antioxidant types, population features, and infertility etiologies—may influence the pooled effect estimates. Despite our rigorous assessments of heterogeneity and robustness, the results of this meta-analysis should be interpreted as an overall therapeutic trend rather than definitive evidence for any single pharmacological intervention. Finally, significant inter-study heterogeneity was observed during the analysis of MII oocyte yields, with the PCOS subgroup being the only cluster to demonstrate minimal intra-group variance—a reflection restricted by the inclusion of only two trials in that subset. Consequently, these specific metrics must be interpreted with caution. Despite these constraints, this study represents a rigorous, comprehensive evaluation of the existing RCT evidence conducted strictly under the PRISMA 2020 guidelines, aiming to provide valuable mechanistic insights and a reference framework for the precise application of antioxidants in assisted reproduction.

## 6 Conclusions

In conclusion, this RCT-exclusive meta-analysis suggests that oral antioxidant supplementation may provide potential clinical benefits in women undergoing IVF/ICSI-ET, particularly by improving the clinical pregnancy rate and MII oocyte yield. Although positive trends were also observed for high-quality embryo counts and live birth rates, these findings are limited by the small number of studies; thus, definitive conclusions cannot be drawn, and further large-scale RCTs are warranted. Exploratory subgroup analyses suggest that the clinical benefits of melatonin may be more consistent and that antioxidants may provide more robust therapeutic effects in patients with PCOS. Furthermore, ovarian reserve status appears to modulate antioxidant efficacy; however, these findings are based on a limited evidence base and should be considered exploratory. In summary, oral antioxidant adjuvant therapy shows potential for improving reproductive outcomes in IVF/ICSI-ET cycles. Future high-quality RCTs, prioritizing live birth and miscarriage rates as primary endpoints, are essential to provide more reliable evidence for the precise application of antioxidants in ART and to inform clinical decision-making.

## Data Availability

The original contributions presented in the study are included in the article/**Supplementary Material**. Further inquiries can be directed to the corresponding author.
